# Denosumab as the Treatment of Recalcitrant Tuberculous Pleural Effusion-Associated Hypercalcemia

**DOI:** 10.1155/2021/5544848

**Published:** 2021-04-19

**Authors:** Afdhal Afiq Abd Jalil, Sharifah Faradila Wan Muhamad Hatta, Aimi Fadilah Mohamad, Mohammed Fauzi Abdul Rani

**Affiliations:** ^1^Department of Internal Medicine, Faculty of Medicine, Universiti Teknologi MARA, Sungai Buloh, Selangor, Malaysia; ^2^Endocrine and Diabetes Unit, Department of Internal Medicine, Faculty of Medicine, Universiti Teknologi MARA, Sungai Buloh, Selangor, Malaysia

## Abstract

Denosumab is a human monoclonal antibody that binds to RANKL (receptor activator of nuclear factor-kappa B ligand). It has mainly been used in the treatment of osteoporosis for a variety of causes especially in situations refractory to bisphosphonates or when kidney function is impaired. It is also used in cases of malignancy-associated hypercalcemia. There are many causes of hypercalcemia, but only rarely it is associated with granulomatous diseases such as tuberculous pleural effusion. We report a case of hypercalcemia from tuberculous pleural effusion that was initially admitted with left medium abundance pleural effusion and a serum corrected calcium level of 3.48 mmol/L. The calcium level was successfully normalized within 72 hours of subcutaneous denosumab administration after other interventions have failed.

## 1. Introduction

Hypercalcemia has often been described in patients with granulomatous disorders, but they are rarely symptomatic [1–4]. Among them, pulmonary tuberculosis is the most common cause but less so among extrapulmonary cases. Locally, the incidence has been reported to be around 27.5% among patients with active tuberculosis [4], which is comparable to the incidence rate in the west or a high TB incidence country such as India [5, 6].

Acute treatment of hypercalcemia from any cause includes intravenous volume repletion or hydration, coupled with bisphosphonate, calcitonin, or denosumab, if the hypercalcemia persists despite hydration. The most suitable choice of treatment depends on the patient's clinical condition, which includes renal function status.

Denosumab is a human monoclonal antibody that inhibits the formation, function, and survival of osteoclasts and reduces bone resorption. It has been used for the treatment of hypercalcemia as an alternative to bisphosphonate, especially in patients with reduced creatinine clearance [7, 8].

We report a case of hypercalcemia secondary to extrapulmonary tuberculosis successfully treated with denosumab after other interventions have failed to reduce the calcium level.

## 2. Case

A 70-year-old male with a known history of type 2 diabetes mellitus, hypertension, chronic kidney disease, and ischemic heart disease presented to the emergency department with 7-day history of gradual onset of shortness of breath, reduced effort tolerance, and lethargy. On further inquiry, there was loss of appetite for a month, but he denied any accompanying weight loss, and there was also no history of fever or night sweats. He admitted to vague abdominal and back pain that lasted for a week.

At the emergency department, he looked cachexic and dehydrated but was alert to time, place, and person. He was apyrexial with a pulse rate of 88 beats per minute and blood pressure of 131/72 mmHg, but tachypnoeic with a respiratory rate of 27 breaths per minute. The oxygen saturation, however, was maintained at 98% on room air. Respiratory examination revealed left-sided stony dullness up to the midzone on percussion with reduced air entry on the same side. Cardiovascular and gastrointestinal examinations were unremarkable.

**Figure 1:**
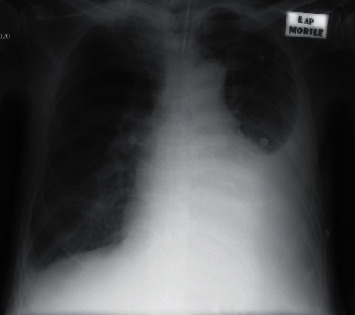
Left medium abundance pleural effusion.

Chest radiograph confirmed the presence of a left medium abundance pleural effusion (Figure 1).

Table 1 summarizes the results of the blood investigations including hypercalcemia with a level of 3.03 mmol/L.

He was initially admitted to the ward for treatment with intravenous Augmentin and 0.9% saline hydration for presumed acute-on-chronic kidney injury as we did not have any baseline kidney function, but unfortunately his breathing deteriorated rapidly a day after admission. He became more tachypnoeic, and the arterial blood gases showed type 2 respiratory failure (pH, 7.32; pO_2_, 62 mmHg; pCO2, 50 mmHg; HCO_3_, 20; lactate, 3). In view of the worsening ventilation and his general medical condition, a decision was made to invasively ventilate him in the intensive care unit (ICU). In the ICU, his antibiotic was upgraded to intravenous piperacillin/tazobactam for presumed community-acquired pneumonia with pleural effusion and intravenous 0.9% saline 1000 mls over 24 hours for hydration was maintained.

Echocardiography demonstrated globally dilated severe hyperkinesia of the left ventricle and a severely impaired systolic function with a left ventricle ejection fraction of 20%. Because of this, the fluid balance was closely monitored to minimize the risk of pulmonary congestion.

His urine output remained good with an average of 100 mls/h equivalent to 1.4 mls/kg/h. Unfortunately, despite adequate hydration and a positive balance of 900 mls averaging over 3 days, his serum calcium continued to rise to 3.48 mmol/L.

Intravenous furosemide was also initiated as a forced diuresis measure, which also failed to bring the calcium down.

Further investigations for the cause of the pleural effusion included therapeutic and diagnostic left thoracocentesis, and blood tests for the cause of hypercalcemia were conducted during admission. The results are listed in Table 2.

To reduce the calcium, we decided to try him on subcutaneous denosumab at the dose of 120 mg, and his serum corrected calcium gradually improved to 2.40 mmol/L, as depicted in Table 3, after 4 days with no further drop in calcium thereafter.

At day 5, the pleural fluid analysis for rapid mycobacterium liquid culture and sensitivity test came back positive for *Mycobacterium tuberculosis* complex. The treatment for tuberculous pleural effusion with isoniazid, rifampicin, pyrazinamide, ethambutol, and pyridoxine (2HRZ/4HR) was subsequently commenced.

He was successfully extubated after 10 days in the ICU and was transferred to the general medical ward for further management. Unfortunately, he developed a severe concomitant hospital-acquired pneumonia complicated by respiratory failure that eventually led to multiorgan failure, cardiorespiratory arrest, and death.

## 3. Discussion

Hypercalcemia can rarely be a manifestation of granulomatous disease including pulmonary tuberculosis [3], even rarer in tuberculous pleural effusion as in our case.

Evaluation of hypercalcemia in our patient was suggestive of a parathyroid-independent mechanism of hypercalcemia with elevated level of vitamin D. In normal situations, vitamin D_3_ is absorbed by the skin from ultraviolet B light which is then metabolised into 25(OH)D_3_ in the liver by 25-hydroxylase. 25(OH)D_3_ is then catalysed via 1-*α*-hydroxylase to an active form known as 1,25-dihydroxyvitamin D_3_ (1,25(OH)_2_D_3_) which aids in calcium absorption in the gut [9].

Tuberculosis patients experience dysregulated extrarenal production of 1,25(OH)_2_D_3_ by activated alveolar macrophages which leads to increased calcium absorption in the gut and thus hypercalcemia [10]. Overproduction of 1,25(OH)_2_D_3_ is a protective mechanism against oxidative injuries due to the nitric oxide burst from granulomatous macrophages [11]. Unfortunately, in some individuals, this protective mechanism has led to a different complication. However, not everyone who suffers from TB would develop hypercalcemia as 1,25(OH)_2_D_3_ is usually produced in small quantities locally and is not brought to target sites for the regulation of calcium homeostasis. This mechanism has been associated with hypercalcemia in pulmonary TB; however, the underlying cause for hypercalcemia in tuberculous pleural effusion, as seen in our patient, remains uncertain.

A theory has been proposed that hypercalcemia in tuberculous pleural effusion could be secondary to circulating monocytes, which act as a source of 1-*α*-hydroxylase that could convert 25(OH)D_3_ to 1,25(OH)_2_D_3_. This 1,25(OH)_2_D_3_ produced by monocytes acts locally and when carried to target tissues, it may play a role in calcium homeostasis, unlike alveolar immune cells which only acts locally [12].

The management of hypercalcemia depends on the level of serum calcium, clinical symptoms, and the underlying disorder. The treatment would always start with adequate hydration and forced diuresis with furosemide followed by bisphosphonates if both measures have failed [13]. Haemodialysis may be required in the event of renal failure and corticosteroids in patients with sarcoidosis or certain neoplasms [14].

In our patient, bisphosphonate was not chosen as the first-line treatment because of the persistently low creatinine clearance (CrCl) of <30 mls/min with a serum creatinine of 212 *μ*mol/L and the worry of further deterioration in renal function. Palmer et al. showed that a quarter of patients with baseline renal dysfunction experienced all-grade serum creatinine elevation when the hypercalcemia was treated with intravenous bisphosphonates. The incidence of serum creatinine elevation was statistically higher in those with a CrCl of <30 mL/min compared to those with CrCl ≥30 mL/min [15]. Calcitonin was also not available at our hospital, hence the decision to use denosumab.

Denosumab is a novel, fully human monoclonal antibody which prevents the receptor activator of nuclear factor-kappa B ligand from binding to its receptor and inhibits osteoclast development, activation, and survival. It has no dose adjustments for renal impairment and has a lower incidence of renal failure and acute phase reactions. It has been approved for the treatment of osteoporosis in postmenopausal women and prevention of skeletal-related events including fractures, spinal cord compression, bone pain requiring surgery/radiation therapy, and hypercalcemia in malignancy [16].

To the best of our knowledge, our case report is the second to report improvement in hypercalcemia secondary to tuberculosis with denosumab. The first case by Torres-Ortiz et al. reported normalization of serum calcium level at 48 hours postadministration and as the patient survived, they were able to observe hypocalcemia at day 16 post-administration of denosumab [17] which is a known side effect of the drug [18]. When compared with zoledronic acid treatment for patients with advanced cancer with bone metastases or myeloma, 5.7% of patients who received denosumab required intravenous calcium infusion compared to 2.7% in the zoledronic acid arm [19]. We were not able to determine if this side effect had occurred in our patient.

During our investigation, ionized calcium could have been done to confirm true hypercalcemia but the presence of two samples of elevated serum total calcium was used instead to indicate true hypercalcemia in our patient.

In conclusion, denosumab is an option for the treatment of refractory hypercalcemia in the setting of low creatinine clearance; however, careful monitoring of calcium levels post-therapy is essential after administration especially in those deficient in vitamin D.

## Figures and Tables

**Table 1:** Baseline blood investigation.

Investigation	Results	Normal values
Haemoglobin	9.3 g/dL	13–17
Total white count	2.48 × 10^^^9/L	4–10
Platelet	167 × 10^^^9/L	150–410
Sodium	134 mmol/L	135–145
Potassium	4 mmol/L	3.5–5.1
Urea	14.10 mmol/L	2.78–8.07
Creatinine	212 *μ*mol/L	62–106
eGFR	26.9 mL/min/1.73 m^2^	>60 mL/min/1.73 m^2^
Magnesium	1.02 mmol/L	0.66–0.99
Corrected calcium	3.03 mmol/L	2.20–2.55
Phosphate	1.33 mmol/L	0.80–1.45
Total protein	83.9 g/L	64.0–83.0
Albumin	30 g/L	35–52
Total bilirubin	20 *μ*mol/L	<21
Alanine transaminase	8 U/L	<41
Alkaline phosphate	146 U/L	40–130
Gamma-glutamyl transferase (GGT)	67 U/L	<60
Erythrocyte sedimentation rate (ESR)	>120	1–15
Full blood picture	Macrocytic anemia: macrocytosis of RBC with polychromatic cells and mild rouleaux formation. No blast or abnormal lymphoid/plasmacytoid looking cell.	

**Table 2:** Further blood samples sent for investigation of hypercalcemia.

Investigations	Results	Normal values
Intact parathyroid hormone	<0.5 pmol/L	1.96–8.49
Total vitamin D/25-hydroxyvitamin D	106.30 nmol/L	50–125
Alpha fetoprotein (AFP)	<1.00 ng/mL	0.89–8.78
CEA	1.37 ng/mL	0.00–5.00
Prostate-specific antigen	0.163 ng/mL	0.00–4.0
Immunoglobulin A (IgA)	4.3 g/L	0.70–4.00
Immunoglobulin G (IgG)	26.3 g/L	7.0–16.0
Immunoglobulin M (IgM)	1.58 g/L	0.40–2.30
Kappa free light chain	352.0 mg/L	6.7–22.4
Lambda free light chain	493.0 mg/L	8.3–27.0
Kappa/lambda ratio	0.71	0.31–1.56
Serum protein electrophoresis	No paraprotein band	
Urine protein electrophoresis	No paraprotein band	
Pleural fluid analysis	Colour	Straw coloured
	LDH (pleural fluid/serum)	Exudative 0.75
	Total protein (pleural fluid/serum)	Exudative 0.7

**Table 3:** Serum calcium level after administration of subcutaneous denosumab.

Day (postdenosumab)	1	2	3	4	5	6	7	8
Corrected calcium level (mmol/L)	3.44	3.43	3.28	2.46	2.97	2.40	2.52	2.43
Serum creatinine level (*μ*mol/L)	353	360	370	273	332	234	345	431

## Data Availability

The clinical data used to support the findings of this study are included within the article.
